# Seronegative granulomatosis with polyangiitis presenting as an acute skull base osteomyelitis with multiple cranial neuropathies

**DOI:** 10.1093/jscr/rjab476

**Published:** 2021-10-20

**Authors:** Andrew Lotfallah, Shayan Shahidi, Sven Wilhelm Odelberg, Adnan Darr, John Mathews

**Affiliations:** Otolaryngology, New Cross Hospital, Wolverhampton, UK; Otolaryngology, New Cross Hospital, Wolverhampton, UK; Otolaryngology, New Cross Hospital, Wolverhampton, UK; Otolaryngology, New Cross Hospital, Wolverhampton, UK; Otolaryngology, New Cross Hospital, Wolverhampton, UK

## Abstract

Granulomatosis with polyangiitis (GPA, formerly Wegener’s) is a rare form of vasculitis, commonly affecting the upper and lower respiratory tract with simultaneous glomerulonephritis. Ear, nose and throat (ENT) manifestations account for the majority of presentations. The presence of antineutrophil cytoplasmic antibody is a recognized hallmark of GPA, but clinicians should remain cautious of false negative results. We describe a rare case of GPA presenting with concurrent middle ear disease and multiple lower cranial nerve palsies. Clinical judgment was affected by repeated negative autoimmune screens, and a definitive diagnosis was only achieved following renal biopsy. Reported cases of GPA presenting with mastoiditis or cranial nerve involvement are typically seropositive, with seronegative GPA following a less aggressive process. This case highlights the importance of clinical suspicion in the face of treatment resistant ENT pathology, and the need for early histopathological analysis. Early diagnosis and treatment are crucial in limiting disease progression.

## INTRODUCTION

Granulomatosis with polyangiitis (GPA, formerly Wegener’s granulomatosis) is a rare autoimmune vasculitis affecting multiple organ systems with significant morbidity and mortality. Diagnosis is based on clinical presentation, positive serology and histopathological analysis. However, an over-reliance on autoimmune screening can lead to delayed diagnosis and initiation of appropriate treatment. Clinically suspected cases should proceed to histopathological evaluation as soon as possible despite negative serology, as we describe in this rare case presenting with concurrent middle ear disease and multiple lower cranial nerve neuropathies.

## CASE HISTORY

A 35-year old, previously fit male presented with an 8-week history of right sided otalgia, intermittent blood-tinged otorrhoea, tinnitus and hearing loss, unresponsive to antibiotics for a presumed otitis media. Two-weeks prior to hospital admission he developed a right sided facial palsy and progressive dysphagia. Past medical history comprised of a right sided mastoidectomy in childhood.

Positive examination findings consisted of a House-Brackmann grade IV facial palsy. Otoscopy demonstrated blood-stained debris and postero-inferior atelectasis. Oropharyngeal and laryngeal examination yielded a right vocal cord palsy combined with both a left sided uvula and right sided tongue deviation associated with wasting. Based on initial assessment, mastoiditis with skull base involvement of the jugular foramen was suspected. The patient was admitted under ear nose and throat (ENT) and commenced on intravenous ceftazidime and gentamicin.

**Figure 1:**
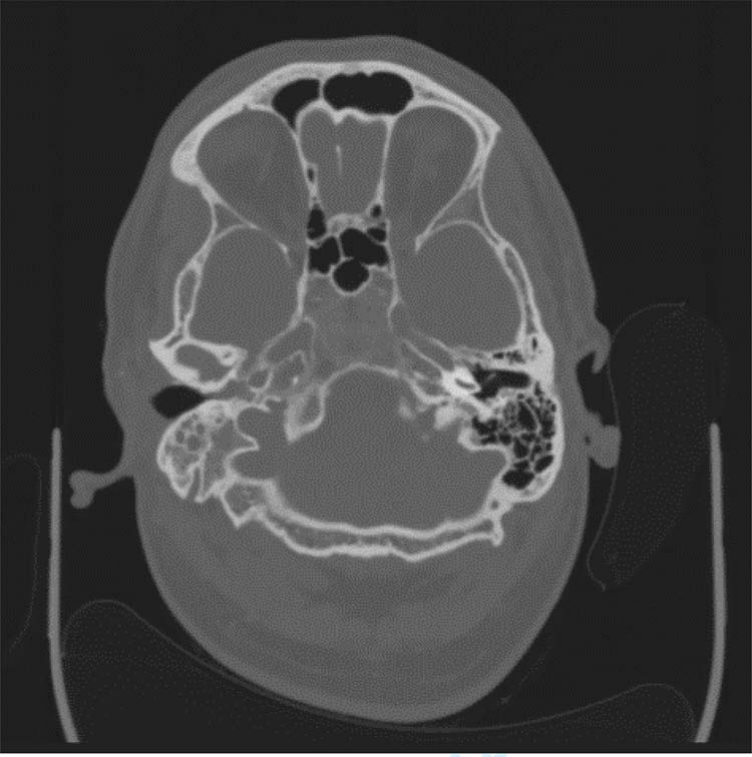
Axial CT image slice showing opacification of the right middle ear cavity and mastoid air cells.

**Figure 2:**
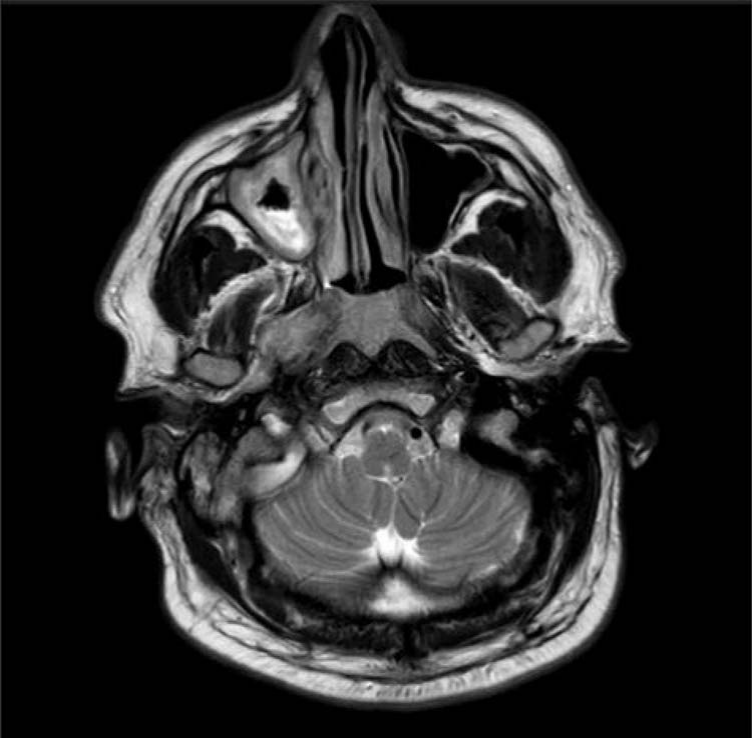
Axial MRI image showing high attenuation lesion affecting the right skull base.

Computerized tomography (CT) of the internal meatuses demonstrated soft tissue thickening of the right middle ear cavity, aditus, antrum and mastoid air cells in keeping with otomastoiditis (Fig. 1), but no destructive or erosive pathology to account for the multiple neuropathies elicited on examination. A subsequent magnetic resonance imaging (MRI) scan demonstrated a 2.5 centimeter (cm) right skull base inflammatory mass, involving the internal carotid artery and jugular foramen (Fig. 2). Anti-microbial therapy was subsequently changed to intravenous meropenem and topical ciprofloxacin ear drops. The patient underwent a right cortical mastoidectomy and grommet insertion at Day 5, with well-pneumatized air cells and inflammatory tissue observed within the aditus.

**Figure 3:**
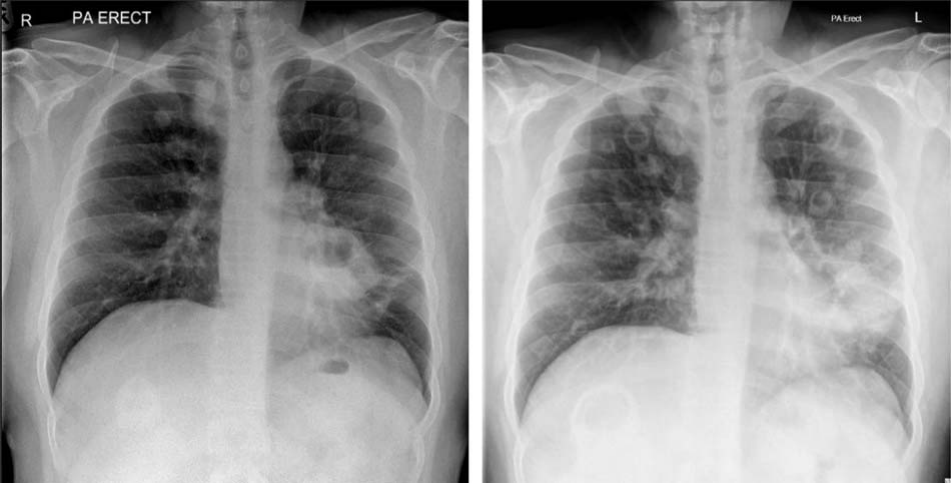
Chest radiographs showing progression of cavitating pulmonary lesions.

Due to persistent otalgia and two episodes of transient loss of consciousness, cross-sectional imaging by way of a CT scan was undertaken, which was unremarkable. An MR venogram however, demonstrated a right sided sigmoid sinus thrombosis, and he was commenced on treatment dose low molecular weight heparin. In conjunction with multiple specialty review and advice, an autoimmune profile was requested, which was negative, as were sputum cultures and bronchial washings for acid-fast bascilli. Due to a sustained pyrexia, multiple blood cultures as well as a trans-thoracic echocardiogram were undertaken to rule out infective endocarditis, which were again negative. Persistently raised inflammatory markers led to a CT thorax–abdomen–pelvis (TAP) request, which demonstrated multiple cavitating pulmonary nodules and widespread mediastinal nodes. Bronchoscopy and biopsy of a left upper lobe thickening merely demonstrated non-specific inflammation. With no improvement clinically, repeat MRI of the head demonstrated progression of the skull base inflammatory mass with extensive soft tissue thickening along the course of the internal carotid artery and pachymeningitis. In addition, repeat chest radiography revealed an increase in size and number of pulmonary nodules (Fig. 3).

At Week 8 following admission, the patient’s clinical predicament worsened, with ongoing hemoptysis, macroscopic hematuria and declining renal function, with an eGFR of 27. A provisional diagnosis of vasculitis was made by the renal physicians, and he was commenced on high dose intravenous steroids. The patient underwent a renal biopsy, with provisional reporting suggesting features consistent with GPA. He was subsequently transferred to a tertiary referral center where he was treated for several weeks with Rituximab, high dose oral steroids, oral anticoagulation and hemodialysis before being deemed fit for discharge. He was referred back to our institution for regular outpatient hemodialysis.

Fortunately, the patient’s vasculitis has since remitted with regular oral steroid therapy and his kidney function has improved, no longer requiring dialysis. However, he continues to suffer symptoms of headaches, dysphagia, poor appetite, weight loss and features of postural hypotension. The patient is undergoing multi-specialty review as an outpatient with input from ENT, ophthalmology, neurology and renal specialists.

## DISCUSSION

GPA is a rare immune-mediated systemic vasculitis characterized by granulomatous lesions [1]. It may impact any organ system, but ear nose and throat (ENT) involvement accounts for ~70% of symptoms [2]. Rhinological disorders comprise the majority of presentations with features of rhinitis, sinusitis, crusting, ulceration and septal perforation [1]. Diagnosis is based on clinical presentation, positive serology and histopathological evaluation [3]. Antineutrophil cytoplasmic antibody (ANCA) is highly specific for cases of GPA, however a false negative result should always be suspected if clinical signs are consistent with a diagnosis [3].

Middle ear disease and mastoiditis have been reported as rare presenting features of GPA, with some cases involving concurrent facial nerve palsy [4]. However, to the best of the author’s knowledge, this is the first reported case involving both middle ear disease and multiple lower cranial nerve pathology as the primary presenting features of GPA. Skull base osteomyelitis was initially suspected, however, a suboptimal response to antibiotic therapy, and manifestation of respiratory and renal pathology increased clinical suspicion of a systemic vasculitis. ANCA negativity led to a degree of uncertainty, which delayed definitive diagnosis and raised the possibility of significant morbidity. Renal biopsy proved to be the only definitive diagnostic investigation.

Without treatment, the mortality of GPA at 1 year is as high as 80%, but this is significantly reduced with intensive immunosuppressive therapy [5]. Otolaryngologists should remain vigilant in cases of treatment resistant pathology, particularly with concurrent systemic decline, despite negative serological investigations. Prompt diagnosis is crucial in initiating timely intervention and limiting disease progression and mortality.
